# Secondary Growth and Carbohydrate Storage Patterns Differ between Sexes in *Juniperus thurifera*

**DOI:** 10.3389/fpls.2016.00723

**Published:** 2016-05-26

**Authors:** Lucía DeSoto, José M. Olano, Vicente Rozas

**Affiliations:** ^1^MedDendro Lab, Centro de Ecologia Funcional, Universidade de CoimbraCoimbra, Portugal; ^2^Área de Botánica, EUI Agrarias, Universidad de ValladolidSoria, Spain; ^3^Facultad de Ciencias Forestales y Recursos Naturales, Universidad Austral de ChileValdivia, Chile

**Keywords:** dioecy, earlywood, latewood, non-soluble sugars, tree growth, sexual dimorphism, soluble sugars

## Abstract

Differences in reproductive costs between male and female plants have been shown to foster sex-related variability in growth and C-storage patterns. The extent to which differential secondary growth in dioecious trees is associated with changes in stem carbohydrate storage patterns, however, has not been fully assessed. We explored the long-term radial growth and the seasonal variation of non-structural carbohydrate (NSC) content in sapwood of 40 males and 40 females *Juniperus thurifera* trees at two sites. NSC content was analyzed bimonthly for 1 year, and tree-ring width was measured for the 1931–2010 period. Sex-related differences in secondary growth and carbohydrate storage were site-dependent. Under less restrictive environmental conditions females grew more and stored more non-soluble sugars than males. Our results reinforce that sex-related differences in growth and resource storage may be a consequence of local adaptation to environmental conditions. Seasonal variation in soluble sugars concentration was opposite to cambial activity, with minima seen during periods of maximal secondary growth, and did not differ between the sexes or sites. Trees with higher stem NSC levels at critical periods showed higher radial growth, suggesting a common mechanism irrespective of site or sex. Sex-related patterns of secondary growth were linked to differences in non-soluble sugars content indicating sex-specific strategies of long-term performance.

## Introduction

In dioecious plant species, female and male individuals frequently display different performance, i.e., sexual dimorphism, within the same population. Sexual dimorphism is the result of individuals differing in life-history, morphological or functional traits depending on their gender (Geber et al., 1999). The theory of sex allocation establishes that females and males may evolve alternative optimal strategies of resource investment in order to maximize reproductive success when resources are limited (Charnov, 1982). Differences in reproductive demands may lead to sex-specific patterns of resource use in dioecious plants (Case and Ashman, 2005). Male reproductive activity is restricted to flowering and pollen dispersal, whereas the female function continues with fecundation, seed and fruit production and dispersal (Lloyd and Webb, 1977). As a consequence, female plants usually allocate proportionally more resources to reproduction leaving less resource available for other functions, such as growth, storage, maintenance or defense, than do males (Nicotra, 1999; Nicotra et al., 2003; Mitchell et al., 2004). Therefore, low performance in females suggests higher reproductive costs (Obeso, 2002). In many dioecious woody plants, males exceed females in size, growth rate or survival (Obeso, 2002; Nuñez et al., 2008; Cedro and Iszkułao, 2011). However, despite higher reproductive costs, females can display equal or higher growth rates (Rovere et al., 2003) due to compensatory mechanisms (Tozawa et al., 2009) and then, sexual dimorphism in growth may be not noticeable.

Dioecious woody plants have shown sex-specific patterns of both water use efficiency and response to water availability. Males are considered as more drought-resistant (Ueno and Seiwa, 2003; Dudley, 2006; Dudley and Galen, 2007; Xu et al., 2008b), while females are more drought-sensitive (Rozas et al., 2009; Bochenek and Eriksen, 2010; Zhang et al., 2010). However, it has also been reported that females may outperform males under well-watered conditions (Ueno and Seiwa, 2003; Dudley, 2006; Hultine et al., 2008; Xu et al., 2008a), and hence, sex-related allocation strategies can be context dependent. For instance, higher male/female ratios have been found under more stressful conditions (Ortiz et al., 2002; Zhang et al., 2010), and shifts in the relative growth rate between males and females have been observed in contrasting environments (Nuñez et al., 2008; Olano et al., 2015). Furthermore, within-sex competition has been associated with reductions in growth rates (Zhang et al., 2009), suggesting niche segregation between sexes. Therefore, sex-related reproductive costs and compensatory physiological mechanisms may also lead to differential performance of males and females under environmental gradients.

Resource allocation to reproduction may differ in terms of timing as well as quantity (Ashman and Baker, 1992; Case and Ashman, 2005). Investment by males is concentrated prior to and at the beginning of the reproductive period, whereas females extend this period with a large proportion of their reproductive resources allocated to subsequent seed and fruit development and ripening (Espírito-Santo et al., 2003). Different timing in resource consumption associated with sex in dioecious woody plants could also result in temporal variation in resource allocation to vegetative growth. Plant growth relies on available carbohydrates, both recently assimilated and stored (Carbone et al., 2013; Richardson et al., 2013). In woody species, during the reactivation of stem cambial activity, stored carbohydrates can be used as the main source of energy; while during xylogenesis a constant supply of sugars from photosynthesis is needed for cell wall synthesis (Oribe et al., 2003). Isotopic analyses reveal a strong linkage between δ^13^C of the earlywood of 1 year and the latewood of the previous year (Vaganov et al., 2009), which has been also confirmed with radiocarbon-based analysis of carbon pools in trees (Carbone et al., 2013). Altogether, these studies suggest xylem growth depends on both recent C-storage and immediately produced carbohydrates. Carbohydrate allocation to growth usually competes with alternative functions, such as maintenance and defense, and it is prioritized over reproduction and storage (Wiley and Helliker, 2012). However, variation in reproductive demands under carbon-limited conditions may lead to a sex-specific reduction in carbohydrate allocation to growth or storage in favor of reproduction (Ida et al., 2015). Moreover, sex-related differences in carbon storage would be also reflected in sex-related growth rates if reproductive C-demands were high.

This study aimed to explore the sex-specific temporal patterns of annual radial growth of xylem, and sapwood C-storage, measured as NSC content in the dioecious conifer *Juniperus thurifera* L. We evaluated the variation of NSC contents over a year and radial growth rates during a 80-years period to test the following hypotheses: (1) sapwood NSC content varies between the sexes in terms of concentration and temporal patterns, because male reproductive function mainly occurs at the beginning of the reproductive period, whereas female function extends during the whole reproductive period till fruit ripening (Espírito-Santo et al., 2003; Montesinos et al., 2012b); (2) radial growth rate is lower in female trees, particularly under more limiting conditions (Obeso, 2002; Montesinos et al., 2006; Rozas et al., 2009; Olano et al., 2015); and (3) secondary growth rates are related to hierarchies in stem NSC contents and this relationship is modulated by sex.

## Materials and Methods

### Study Species

*Juniperus thurifera* L. (Cupressaceae, Spanish juniper) is an evergreen long-living dioecious conifer, typically found in continental areas of the Western Mediterranean Basin at elevations ranging from 200 to 3,400 m with its largest populations in Spain and Morocco (ca. 200,000 and 30,000 ha, respectively; Gauquelin et al., 1999; Blanco et al., 2005; DeSoto et al., 2012). The continental Mediterranean climate is characterized by dry summers and cold winters, constraining *J. thurifera* secondary growth to the favorable periods in spring and early autumn (Camarero et al., 2010). Contrastingly, its photosynthetic activity is maintained all year round, but at with low rates during winter (Gimeno et al., 2012; Esteban et al., 2014). Flowering occurs in late winter and ripening of female cones takes almost 2 years (Amaral-Franco, 1986). It usually displays sex-specific physiological traits, with females showing higher photosynthetic rate and stomatal conductance than males (Montesinos et al., 2012b). Previous studies also showed asymmetric growth patterns and climatic sensitivities according to sex and age (Montesinos et al., 2006; Rozas et al., 2009; Olano et al., 2015) and higher reproductive costs for females (Montesinos et al., 2012a).

### Study Sites

**FIGURE 1:**
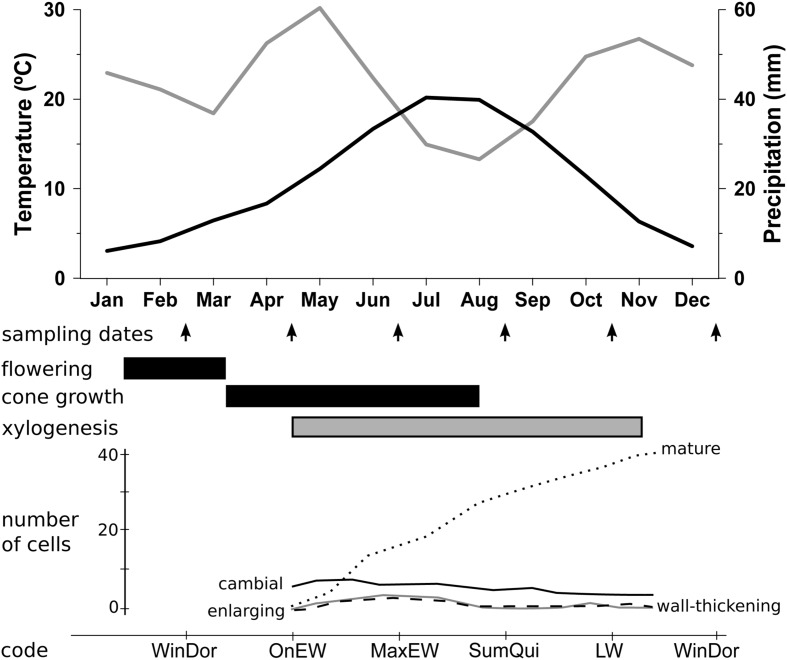
**Monthly variation of climatic conditions and phenological stages.** Climate diagram of monthly precipitation (gray line) and monthly mean temperature (black line) for the period 1961–2010 from the Soria meteorological station (41° 46′ N, 02° 28’ W, 1,082 m elevation, 30 km from the study site). Arrows indicate sampling dates and lower bars indicate main reproductive (black) and vegetative (gray) phases. Number of cells in developmental phases, cambial cells (solid black line), radially enlarging tracheids (solid gray line), wall-thickening and lignification tracheids (dashed line) and mature tracheids (doted line) are also showed (see Figure 3 in Camarero et al., 2010). Codes of xylogenesis periods indicate the correspondence between sampling dates and vegetative phases: LW, LW growth; MaxEW, Maximum of EW growth; OnEW, Onset of EW growth; SumQui, Summer Quiescence; WinDor, Winter Dormancy.

The study area was located in the Cabrejas range, near the village of Cabrejas del Pinar (41° 47′ N, 2° 50′ W), Soria Province, Spain (see Supplementary Figure S1). The climate is subhumid supramediterranean (Rivas-Martínez and Loidi, 1999), with a mean annual rainfall of 533 mm and mean monthly temperatures ranging from 2.8°C in January to 20.0°C in July (**Figure 1**). Summer drought lasts 2 months, from July to August, but negative water balance occurs from May to September (DeSoto et al., 2014). Two 1-ha plots at 1,200 m elevation were sampled in the Cabrejas range as replicates for this study. Site A was located on a plateau, whereas Site B was located on a north-facing slope. In Site-A, tree density was 315 trees ha^-1^ for *J. thurifera*, with a minor presence (20 trees ha^-1^) of *Quercus ilex* L. subsp. *ballota* (Desf.) Sam and *Pinus sylvestris* L. The understory is dominated by xerophytic grasses and shrubs including *Festuca hystrix* Boiss., *Cistus laurifolius* L., *Lavandula latifolia* Medicus, and *Satureja intricata* Lange. In Site-B, tree density was 375 trees ha^-1^ for *J. thurifera*, and 90 trees ha^-1^ for *Q. ilex*, with similar understory species diversity but also *Arctostaphylos uva-ursi* (L.) Sprengel patches (see Supplementary Figure S1). Number of females and males did not depart from 1:1 sex ratio in both study sites (Site-A: N_female_ = 127, N_male_ = 156, N_non-sexed_ = 32; χ^2^ = 2.97, *p* = 0.09; Site-B: N_female_ = 179, N_male_ = 160, N_non-sexed_ = 36; χ^2^ = 1.06, *p* = 0.32). Spatial point pattern analysis of trees position did not find any evidence of spatial segregation between sexes (DeSoto et al., 2010).

### Sample Collection

We randomly selected 40 dominant trees (20 males and 20 females) per site and measured individual height and DBH (diameter at breast height, 1.3 m; see Supplementary Table S1). For the analysis of NSC content, one wood core of 0.5 cm diameter and 8 cm length per tree was extracted bimonthly, between June 27th 2006 and June 28th 2007 (seven sampling dates, 560 cores in total), following a spiral around the trunk starting at ~1.3 m of height. The sampling dates were selected in accordance to the phenological stages of radial increment growth revealed by micro-core preliminary analysis from a simultaneous study within the same stand (Camarero et al., 2010, **Figure 1**). The reproductive phases of *J. thurifera* were estimated by a 3-years period of field observations (E. Rodríguez personal communication, **Figure 1**). We measured sapwood width on the seven cores collected from each tree, considering this a surrogate measure of the potential area of sap conduction. After collection, wood samples were placed in a dark cooler in the field and immediately stored at -18°C in a laboratory freezer until chemical analysis was done. In 2011, two wood cores per tree were collected at a height of 1.3 m for tree-ring measuring and age estimation.

### Carbohydrates Analysis

Sapwood cores collected for chemical analyses were oven-dried at 60°C for 3 days. Bark was removed and the outermost 2 cm of sapwood selected, where highest xylem NSC concentration is found (Hoch et al., 2003). Although, phloem has higher NSC concentrations than xylem, stem sapwood is by far the largest tree NSC reservoir due to its greater volume (Simard et al., 2013). Samples were finely ground prior to chemical analysis. The soluble and non-soluble fractions of NSC were calculated using the perchloric acid/anthrone method (Morris, 1948; Olano et al., 2006; see Supplementary Methods 1), on 20 mg of dried and powdered sapwood. This procedure distinguishes soluble and non-soluble fractions of NSC, namely SSs, i.e., mono- and disaccharides such as glucose, fructose and sucrose, and NSSs, i.e., polysaccharides mainly starch (Rose et al., 1991). Analysis results were expressed as the percentage (w/w of dry mass) of carbohydrates. The sum of SS and NSS, expressed in glucose equivalents, is referred to as total content of NSC.

### Dendrochronological Analysis

Cores were air-dried, glued onto wooden mounts and polished with a series of successively finer sandpaper grits. Tree-rings were visually dated following a standard procedure (Stokes and Smiley, 1996). We measured EW and LW widths separately with a 0.001 mm resolution, using a Velmex sliding-stage micrometer (Bloomfield, NY, USA) interfaced with a computer. EW and LW were differentiated using a stereo microscope, considering the LW tracheids when lumen was approximately smaller than twice the cell wall (Denne, 1988; DeSoto et al., 2011). Total ring-width (TR) was calculated as the sum of EW and LW for every ring. Measurement and dating errors on individual tree-ring series were detected on TR series with the COFECHA program (Grissino-Mayer, 2001). Cross-dating was performed against a robust master chronology, previously obtained from stem disks of more than 100 trees in the study area (Rozas et al., 2009). Only those ring-width series correctly synchronized were retained for subsequent analyses (*N* = 74). Tree age was individually estimated at breast height.

### Statistical Analysis

To assess whether time, site and sex influenced NSC content in sapwood, we carried out mixed linear models. This approach allows modeling repeated measures on a subject, specifying *a priori* the covariance matrix as an unstructured matrix because the correlations between the pairs of observations were variable and estimated from the data (Quinn and Keough, 2002). We considered the content of SS, NSS, and NSC as response variables, and assumed a normal error distribution with an identity link function for all variables. Models included time, site and sex and all the possible two-way interactions among them as explanatory fixed factors, and sapwood width and tree size (DBH × height) as covariates. Tree (*N* = 80) was considered as a random subject effect.

In order to study the effects of site and sex on ring widths, we used generalized estimating equations (GEEs) via restricted maximum-likelihood (REML) that are an extension of generalized linear models (GLMs) to model correlated data (Quinn and Keough, 2002). GEEs provide a flexible way to model traits which do not satisfy the assumptions of a standard linear modeling and allow the problems arising from data imbalance and repeated structure to be overcome. We specified *a priori* the covariance matrix as an autoregressive structure because it corresponded to a repeated, not balanced, analysis design. Models included site and sex and their interaction as fixed factors, and tree age, sapwood width and year as covariates; core nested in tree (*N* = 74) was considered as the subject effect. We assumed a normal error distribution with an identity link function for log-transformed EW, LW, and TR widths. We fitted mixed linear models and GLMs using the MIXED and the GEMMOD procedures of SAS (SAS Statistical package 9.1, SAS Institute, Raleigh, NC, USA). Differences between least-squares means were tested pairwise by using the DIFF option in the LSMEANS statement.

A linear model was performed to explore whether hierarchies in NSC levels at different times along the year could predict EW width in 2007 and whether that relation was modulated by sex or site. As potential predictors we included SS and NSS values from December to June (8 parameters), as well as site and sex that were included as fixed factors. Best model was selected following Bayesian information criterion (BIC; Zuur et al., 2009). Linear models were performed in R using package *leaps* (R Core Team, R Foundation for Statistical Computing, Vienna, Austria).

## Results

### Sexual Dimorphism in Carbohydrate Content

**FIGURE 2:**
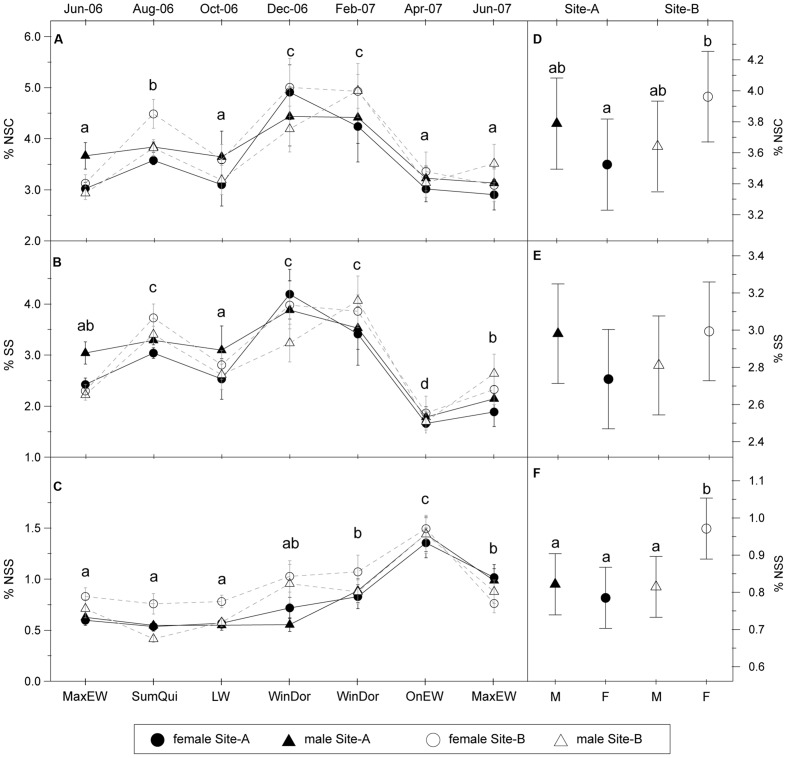
**Seasonal pattern of non-structural carbohydrates.** Left: Seasonal variations of **(A)** total NSC, **(B)** SS, and **(C)** NSS concentration (% of dry weight, mean ± SE) in stem sapwood for each site and sex. Codes of the xylogenesis periods are indicated in **Figure 1**. Right: Model-adjusted least-square means ± 95% confident intervals for the total NSC **(D)**, SS **(E)** and NSS **(F)** concentration (% of dry weight) in stem sapwood during the studying period based on mixed linear model (**Table 2**). Different letters indicate significant differences of Least Square means among sample dates or between sexes and sites (*P* < 0.05; *N* = 40 individuals per site, 20 females and 20 males).

Mean yearly NSC concentration was 3.6%, showing a highly significant seasonal variation (**Table 1**, **Figure 2A**). NSC concentration displayed a maximum (4.5%) in December–February during winter dormancy, and then a spring decrease (3.2%) in both April and June coinciding with the onset and the maximum rate of EW growth, respectively. NSC concentration reached a secondary maximum (3.9%) in August corresponding to summer growth quiescence, and decreased again (3.4%) in October during LW growth period. Mean SS concentration was 2.8% and also showed highly significant seasonal variation (**Table 1**, **Figure 2B**). The pattern was quite similar to that of NSC, with minimum values in April (1.8%), June (2.2% in June 2006; 2.5% in June 2007) and October (2.8%), and two maxima in December–February (4.0%), and August (3.4%).

**Table 1:** Factors affecting non-structural carbohydrate levels.

	df num	df den	NSC		SS		NSS	
Factor			*F*	*P*	*F*	*P*	*F*	*P*
Time	6	457	**16.67**	**<0.001**	**38.28**	**<0.001**	**24.88**	**<0.001**
Sex	1	75	0.03	0.856	0.05	0.827	1.86	0.177
Site	1	75	0.88	0.352	0.09	0.760	**4.11**	**0.046**
Site × Sex	1	75	**5.87**	**0.018**	3.69	0.058	**9.83**	**0.002**
Site × Time	6	457	**2.67**	**0.015**	**4.96**	**<0.001**	1.96	0.070
Sex × Time	6	457	1.19	0.309	0.88	0.510	0.66	0.682
Sapwood	1	457	1.54	0.215	1.65	0.199	2.34	0.127
Tree size	1	75	**(+) 4.33**	**0.041**	**(+) 5.51**	**0.022**	0.02	0.891

*Results of Type III fixed effects test of the mixed linear model for levels of total NSC, SS, and NSS. Data represent the degrees of freedom (df num, df den), the *F*-statistic with the associated *P*-value of significance (bold type for significant effects, *P* < 0.05; N_*Female*_ = 20, N_*Male*_ = 20, for each site) for each factor (time, sex, and site) and their interactions. Tree size (height × DBH) and sapwood width were included in the model as covariates. The signs indicate the direction of the effects.*

Time exerted a strong effect on SS and NSC concentration; however, we did not find significant effects of sex or site (**Table 1**). The interaction term “site × time” was significant for SS and NSC concentration revealing different temporal patterns for each site (**Table 1**), but not for each sex since “sex × time” interaction was non-significant. Finally, we found the interaction term “site × sex” was significant for NSC (and marginally significant for SS), with females showing higher NSC concentrations in the Site-B than females in the Site-A, while concentrations in males did not differ between the sites (**Table 1**, **Figures 2D,E**). SS and NSC concentration showed a significant positive effect of tree size, with greater carbon concentration in larger trees, while the effect of sapwood width on carbohydrate content was not significant (**Table 1**).

Non-soluble sugar accounted for a lower portion of NSC than SS, with an annual mean concentration of 0.8%. NSS also showed significant seasonal variation (**Table 1**) but with a different temporal pattern from SS or NSC pattern. NSS gradually increased during winter and reached a maximum value of 1.4% in April (**Figure 2C**), with lower values between June and December (0.7%). Site had a significant effect with trees in the Site-B having higher NSS levels. The interaction “site × sex” significantly influenced NSS concentrations (**Table 1**), with higher NSS contents occurring in females in the Site-B (1.0%), as compared with females on Site-A and males in both sites (0.8%; **Figure 2F**). All other studied factors showed no significant effects on NSS content.

### Sexual Dimorphism in Growth

**FIGURE 3:**
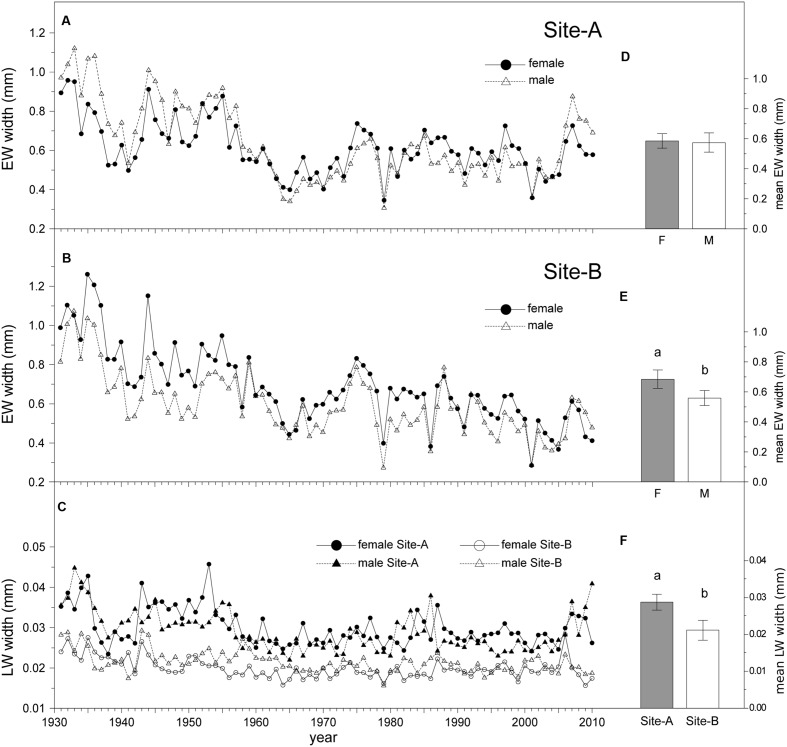
**Long-term radial growth variation.** Left: EW mean ring-width chronologies of *Juniperus thurifera* in **(A)** Site-A, **(B)** Site-B, and **(C)** LW mean ring-width in both sites. **Right:** Model-adjusted back-transformed least-square means ± 95% confident intervals for EW width of different sexes for **(D)** Site-A, **(E)** Site-B, and **(F)** LW width of different sites based on GEE model (**Table 2**). Significant differences of Least-Square means among sample dates or between sexes and sites are indicated with different letters. (*P* < 0.05; N_Female_ = 19; N_Male_ = 18, for each site).

Growth rates of males and females significantly differed (**Table 2**). Mean EW width in female trees (0.63 mm) was greater than in males (0.57 mm) over the period 1931–2010. Site had no effect on EW width, although differences between the sexes were only significant in Site-B, with wider EW in females than in males (**Figure 3**). Trees growing on Site-A showed wider LW than trees on Site-B (**Table 2**, **Figure 3**). The interaction term “site × sex” had no significant effect on growth rates. TR, EW, and LW widths decreased with age. Sapwood width exerted a significant positive effect on both EW and TR widths (**Table 2**).

**Table 2:** Factors affecting secondary growth.

		TR		EW		LW	
Factor	df	χ^2^	*P*	χ^2^	*P*	χ^2^	*P*
Year	1	**(-) 69.66**	**<0.001**	**(-) 59.40**	**<0.001**	**(-) 16.32**	**<0.001**
Sex	1	3.28	0.070	**5.52**	**0.019**	0.15	0.702
Site	1	0.04	0.837	1.90	0.168	**14.28**	**<0.001**
Sex × site	1	3.17	0.075	3.67	0.055	0.46	0.498
Sapwood	1	**(+) 8.17**	**0.004**	**(+) 8.19**	**0.004**	0.59	0.444
Tree age	1	**(-) 29.94**	**<0.001**	**(–) 26.06**	**<0.001**	**(-) 7.68**	**0.006**

*Results of Type III test conducted on the GEE model for the fixed factors year, site and sex and the interactions between them, sapwood width, tree size and tree age, on the EW, LW, and total tree-ring (TR) log-width for the period 1931–2010. Data represent degrees of freedom (df), the χ^2^ and *P*-value of significance (bold type for significant effects, *P* < 0.05; N_Female_ = 19, N_Male_ = 18, for each site). The signs indicate the direction of the effects.*

### Relationship between Hierarchies in NSC Levels and Secondary Growth Rates

Best model explaining EW width in 2007 included positive effects of stem NSS content in previous February (estimate = 0.23 ± 0.07) and June SS content (estimate = 0.06 ± 0.02; *n* = 78, *r*^2^ = 0.18, *P* < 0.001; **Table 3**). Difference in BIC with next model was larger than 2 (3.2), denoting clear difference between models. Sex and site were not included in the best model, thus indicating that the statistical relationship between the NSC hierarchy and EW growth was unrelated to sex or site. The achieved models including NSC levels were substantially better than a model without any factor (**Table 3**).

**Table 3:** Linear model construction relating NSC levels, sex and site with earlywood growth in 2007.

Model	BIC	ΔBIC	NPar
NSS-Feb + SS-Jun	103.781		2
NSS-Feb	107.005	3.224	1
NSS-Feb + SS-Jun + SS-Feb	107.008	3.227	3
NSS-Feb + SS-Jun + sex	107.409	3.628	3
SS-Jun	108.909	5.128	1
NSS-Feb + SS-Feb	109.798	6.017	2
NSS-Feb + site	110.504	6.723	2
No factor (null model)	111.443	7.662	0
Full model	134.367	30.586	10

*Best models are shown. Model selection was done following the Bayesian Information Criterion (BIC). ΔBIC, increment on BIC values respect to that of the model with lowest BIC; NPar, number of parameters in the model. Full model and null model (just constant) are included at the end of the list for comparison. Parameters names are built including NSS and SS, plus month of sampling (Feb, February; Jun, June).*

## Discussion

### Females Grow More and Accumulate More NSC under Less Stressful Conditions

Female trees showed wider EW and had larger NSS (starch) reserves than male trees on Site-B, while neither growth nor NSS concentration showed sex-related differences on Site-A. A plausible explanation can be related to the existence of context-dependent growth responses between male and female *J. thurifera* trees (Olano et al., 2015). Site-A is a plateau with high exposure to wind and radiation, and shallow soil with exposed patches of the limestone bedrock. In contrast, the Site-B is a northern-slope characterized by lower incident radiation and deeper and moister soil. Therefore, *J. thurifera* females performed better than males under more favorable conditions of site-B, but no differences between sexes were found in a more stressful environment.

Our results do not fit with the assumption of the cost of reproduction hypothesis for dioecious woody species, which postulates that the higher reproductive effort of females results into lower resource allocation to the vegetative functions of growth, storage and defense (Obeso, 2002; Espírito-Santo et al., 2003; Massei et al., 2006). *J. thurifera* females can allocate three times more resources to reproduction than males (Montesinos et al., 2012a); however, females can increase their photosynthetic structures after nutrient and water addition to compensate higher reproductive costs, while males cannot (Montesinos et al., 2012b). This sex-related variation in physiological and architectural traits implies that females may show higher performance under favorable environmental conditions (Hultine et al., 2008; Olano et al., 2015), by increasing both carbohydrate production and storage.

Previous studies on dioecious trees have described greater photosynthetic capacities and larger growth rates in female trees under well-watered conditions (Ueno and Seiwa, 2003; Dudley, 2006; Hultine et al., 2008; Xu et al., 2008a). On the other hand, female trees usually show lower water use efficiency than males, which may enhance their sensitivity to drought stress (He et al., 2003; Dawson et al., 2004; Dudley and Galen, 2007; Hultine et al., 2013). In our study, *J. thurifera* females on the mesic Site-B might have benefited from the higher water supply, enabling increased photosynthetic rates (Montesinos et al., 2012b), favoring carbon gain and consequently radial growth. Moreover, it has been also reported that *J. thurifera* males show higher growth than females in semiarid environments where males displayed lower gas exchange rates and higher water use efficiency (Montesinos et al., 2006, 2012b; Olano et al., 2015). We found similar sensitivity of EW growth to climate in both sexes on the dry Site-B, while EW growth of female trees on the mesic Site-B were slightly less sensitive than EW growth of male trees to water availably in May and June (see Supplementary Figure S2). This difference might imply a better performance of females than males in years with early summer drought under the more favorable conditions of site-B, and suggests the sex-specific growth responses of *J. thurifera* trees depending on the local environmental conditions. It is worth noting that differences between males and females could not be attributed to spatial niche segregation (Hughes et al., 2010; Zhang et al., 2010), since trees of both sexes were not spatially segregated in our study sites (unpublished data).

*Juniperus thurifera* is reported as a masting species, i.e., flower and cone production varies between years, with one mast seeding year each 6-years period (Montesinos et al., 2012a), and 2006–2007 was a non-masting period for the species in the study area (Tellería et al., 2011). Carbon allocation priority might only limit female growth during seed masting years since trees preferentially use photosynthates produced in the current year for seed production (Hoch et al., 2013; Ichie et al., 2013). Therefore, under favorable conditions, *J. thurifera* females may increase both stored starch and radial growth during non-masting years when female reproductive costs are reduced. Nevertheless, we did not find higher female EW growth in 2007, probably as a result of tree aging that mitigates sexual dimorphism in growth. *J. thurifera* females showed higher growth rates only when trees younger than 101 years were compared (Rozas et al., 2009), and the sampled trees were around this age (see Supplementary Table S1). Further investigation is needed to understand how patterns of reproductive cost may be reflected on long-term radial growth and whether females may differentially allocate carbon to growth during non-masting periods.

### Seasonal NSC Concentration is Unrelated to Sex and Opposite to Radial Growth Phenology

Non-structural carbohydrate concentrations showed strong intra-annual variability in *J. thurifera*. Seasonal variation of NSC fractions in the stem was unrelated to sex, even under the expectation that female and male functions show different seasonal requirements (Case and Ashman, 2005). Two plausible explanations for this finding can be considered. First, no differences in stem sapwood NSC concentration between flowering and non-flowering seasons were found in previous studies (Newell et al., 2002; Yee and Tissue, 2005). Carbohydrates are prone to flow between the shortest source-sink distances, and consequently, sex-related seasonal NSC storage and mobilization patterns might be only detectable in the sapwood of small branches close to reproductive structures (Matsuyama and Sakimoto, 2008). Second, *J. thurifera* females bear several cohorts of cones at the same time, which demand different quantity and type of resources (Montesinos et al., 2012a). We found more NSS content in female trees under the favorable environment Site-B, but we did not detect evidences of carbon limitation associated with reproductive seasonality. Third, the sampling years were non-masting, potentially reducing the sexual differences in reproductive allocation (Montesinos et al., 2012a). Nevertheless, carbohydrates stored in the main stem, far from reproductive organs, may not be significant contributors to seasonal reproductive demands, and consequently intra-annual variation in the sapwood carbon reserve might be more influenced by other physiological processes, such as photosynthesis, respiration, osmotic regulation or secondary growth, than by reproduction (Dietze et al., 2014; Schiestl-Aalto et al., 2015).

Soluble sugars were the major contributors to total NSC, driving its seasonal pattern. SS concentrations showed a bimodal pattern with minima in spring and autumn and maxima in summer and winter. SS minima closely mimicked the periods of highest cambial activity, April–June and September–October, whereas maxima occurred during summer (August) and winter, when xylogenesis is restrained (Camarero et al., 2010). Presence of maximum SS levels in winter or summer has been reported for other evergreen trees (e.g., Meletiou-Christou et al., 1998; Körner, 2003; Sudachkova et al., 2004; Oberhuber et al., 2011). In fact, carbon gain in evergreen woody species can be achieved all year round, albeit at reduced rates during the unfavorable seasons (Schaberg et al., 1998; Larcher, 2000; Oberhuber et al., 2011). A positive photosynthetic balance may drive the accumulation of SS in *J. thurifera* sapwood during winter and mid-summer, when photosynthesis, but not secondary growth, is active (Larcher, 2000; Körner, 2003; Esteban et al., 2014). Maximum SS levels in winter may be also a consequence of the decrease in parenchyma respiration because of the reduction of enzymatic activity with cold temperatures (Matyssek et al., 2002). However, xylem respiration could not explain the summer SS maximum, suggesting the prioritization of carbon investment in secondary growth over storage in sapwood (Wiley and Helliker, 2012).

Non-soluble sugars concentration in *J. thurifera* sapwood showed low levels during most of the year and displayed an absolute maximum in late April which is consistent with the observations reported for other conifers (e.g., Schaberg et al., 2000; Hoch et al., 2003; Michelot et al., 2012) and also for other evergreen Mediterranean plants (e.g., Cruz and Moreno, 2001; Palacio et al., 2007). Spring NSS accumulation in Mediterranean woody plants has been interpreted as a strategy to overcome future demands, such as the stress caused by summer drought (Meletiou-Christou et al., 1998; Palacio et al., 2007; Piper, 2011), and may constitute an adaptive response to drought-induced carbon depletion (Bréda et al., 2006; Galiano et al., 2011; Wiley and Helliker, 2012). The strategy of reserving starch for overcoming drought could be also a plausible explanation for the starch peak of alpine conifers in April, because drought risk and xylem hydraulic failure not only occur under arid conditions but also in wet woodlands, where stored starch may aid to lessen it (Choat et al., 2012; Mayr et al., 2014).

### Hierarchies in NSC Levels in Stem Can Predict Secondary Growth

Trees with higher stem carbon concentrations at critical periods of the year showed higher growth rates irrespective of site and sex. Thus, NSC pools were a good predictor of subsequent secondary growth. The timing of the statistical links between NSC and tree-ring growth is in agreement with well-known climate-growth relationships in *J. thurifera* (Rozas et al., 2009; Camarero et al., 2010; DeSoto et al., 2012, 2014; Olano et al., 2012, 2015; Rozas and Olano, 2013). Linkage between NSC and EW growth was also consistent with the relationships between climate and EW growth observed in our sampled sites, where EW growths of both female and male trees were mainly affected by winter precipitation and summer drought (see Supplementary Figure S2). The negative influence of winter rainfall on *J. thurifera* growth has been interpreted as a consequence of cloudy/snowy conditions or flooding limiting winter photosynthetic activity, thereby reducing NSC contents (Rozas et al., 2009; DeSoto et al., 2012, 2014; Gimeno et al., 2012; Olano et al., 2012). The amount of NSS accumulated during winter may be converted into mobile C-compounds during reactivation of cambial activity, fostering EW growth (Oribe et al., 2003; Vaganov et al., 2009), and this is consistent with the positive relationship we found between NSS content in February and EW width. Water availability in June is critical for *J. thurifera* EW formation (Rozas et al., 2009; DeSoto et al., 2012, 2014; Olano et al., 2012) as it affects xylem formation through influencing the cambial division rate, cell expansion and wall thickening (DeSoto et al., 2011; Olano et al., 2014). Indeed, EW cell walls show a strong isotopic signal related to June rainfall, indicating that carbon assimilated in June is incorporated into xylem (Olano et al., 2014). Consistently, trees with higher stem SS concentrations in June when cambial activity is maximal (Camarero et al., 2010; Carbone et al., 2013) had higher radial growth rates. Our results suggest that the hierarchies in stored NSC in stems might be a good predictor of secondary growth in *J. thurifera*. However, our study was focused on a single non-masting year’s data and future investigation is needed to address whether the relationship is sustained over time and how reproductive requirements modulate it.

## Conclusion

This study clearly shows sexual dimorphism in C-storage and long-term patterns of growth, but no sexual differences in stem NSC seasonal patterns; this suggests their independence from reproductive phenology. Secondary growth may be prioritized over SS storage in sapwood when cambium is active, while NSS storage in spring may constitute an adaptive response to feasible drought-induced carbon depletion. Regarding sexual dimorphism, sex-specific differences in functional mechanisms for photosynthetic assimilation may allow dioecious trees to compensate for reproductive costs and may cause the sexual differentiation of carbohydrate storage and growth. This response was noticeable under favorable environments where female trees are more able to benefit from non-limiting water supply, promoting higher carbohydrate levels and larger growth rates. Since sex-specific responses seem to be mediated by water availability, and considering that most climate change models predict an increasing incidence of drought in the Mediterranean region, further research in dioecious trees should focus on evaluating how the sexes will respond differentially to these novel scenarios.

## Author Contributions

All the authors conceived the study, designed the sampling and wrote the manuscript. LD measured the samples. LD and JO analyzed the data.

## Conflict of Interest Statement

The authors declare that the research was conducted in the absence of any commercial or financial relationships that could be construed as a potential conflict of interest.

## Abbreviations

EW: earlywood
LW: latewood
NSC: non-structural carbohydrates
NSS: non-soluble sugars
SS: soluble sugars
TR: total tree ring.
