# Supplementary material for: Secondary Growth and Carbohydrate Storage Patterns Differ between Sexes in Juniperus thurifera
Source: Front Plant Sci. 2016 May 26;7:723. doi: 10.3389/fpls.2016.00723 (PMC4880588; doi:10.3389/fpls.2016.00723)
Supplement: Supplementary file 1 [file Table_1.PDF]

frontiers

Supplementary Material

Secondary growth and carbohydrate storage patterns differ between sexes in *Juniperus thurifera*

Lucía DeSoto*, José M. Olano, Vicente Rozas

*** Correspondence:** Corresponding Author: luciadesoto@gmail.com

1 Supplementary Methods

1.1 Carbohydrate analysis: Perchloric acid / Anthrone method

The anthrone technique has been frequently used to analyze Soluble Sugars (SS) and Non-Soluble Sugars (NSS) contents in sapwood (i.e. Cerasoli et al. 2004, Olano et al. 2006). Soluble Sugars (SS) of the powdered sapwood were extracted with 1 ml of ethanol (80 %) at 80 °C during 30 min. and then centrifuged at 3636 g for 10 min. The supernatant was kept for quantification. The process was repeated twice. Finally, 100 µl of the supernatant were combined with anthrone reagent (1 ml) and heated at 100 °C for 15 min. The absorbance of this solution was measured at 630 nm and these values were then regressed against readings from a set of standard solutions of glucose to obtain sugar content. The NSS was extracted after hydrolyzing the residue with 1 ml of perchloric acid (35%) for 1 h. The solubilized NSS solution was then reacted with a mixture of concentrated sulfuric acid and anthrone to hydrolyze NSS to glucose. The extract was then centrifuged at 3636 g for 10 min and the supernatant was used for glucose quantification. The solubilized carbohydrates were analyzed using the anthrone reaction with the approach previously described for SS but with different standard solution. Results were expressed as the percentage (w/w of dry mass) of non-structural carbohydrates contents. The sum of SS and NSS, expressed in glucose equivalents, is referred to as total non-structural carbohydrates (NSC).

1.2 Climate-growth relationship

We used the TS3.23 dataset produced by the Climate Research Unit (CRU 2015, Mitchell and Jones 2005) that provides high-resolution (0.5°×0.5°) grids of monthly climatic variables for the period of 1901–2014 (data downloaded from Climate Explorer of the Royal Netherlands Meteorological Institute, http://climexp.knmi.nl). We downloaded monthly mean temperature and precipitation variables of the CRU grid points nearest the sampling area.
The raw data from the EW and LW growth series correctly synchronized within each site and sex combination were standardized with the R-package *dplR* (Bunn 2010; R

Core Team 2015). The growth series were first fit to a cubic smoothing spline with 55-year period, which was flexible enough to preserve high-frequency climatic information by reducing the non-climatic variance such as age/size trends (Cook and Kairiukstis 1990). For each site and sex, the obtained indices were averaged on a year-by-year basis using a bi-weight robust mean, and the mean series were subjected to autoregressive modeling to obtain residual chronologies of pre-whitened growth indices.
A temporal window of 13 months was selected to identify limiting climatic factors from September of the previous year to September of the year of tree-ring formation for EW series and of 5 months from July to November of the year of tree-ring formation for LW series. Pearson's correlations between residual chronologies for each category of site and sex and the climatic variables were calculated for the common period 1961–2010. We selected this time period because climate-growth relationship shifted in the middle of the 20th century in Soria population (DeSoto et al. 2010, DeSoto et al. 2014). The climatic variables that significantly correlated with the residual chronologies were selected to test the climatic response for each site and sex combination by using General Linear Mixed Models (GLMMs). Models included climatic variables as fixed effects and core nested in tree (N = 74) as random effects. Models were fitted with the R-package *lme4* (Bates et al. 2011; R Core Team 2015).

References not included in the main text:

Bates D, Maechler M, Bolker B (2011) lme4: Linear mixed-effects models using S4 classes. R package version 1.1-10. Available from http://CRAN.R-project.org/package=lme4 .

Bunn AG (2008) A dendrochronology program library in R (dplR). Dendrochronologia 26:115–124.

Cerasoli S, Maillard P, Scartazza A, Brugnoli E, Chaves MM, Pereira JS (2004) Carbon and nitrogen winter storage and remobilisation during seasonal flush growth in two-year-old cork oak (*Quercus suber* L.) saplings. Annals of Forest Science 61: 721– 729.

Cook ER, Kairiukstis L (1990) Methods of dendrochronology: applications in the environmental sciences. Kluwer Academic Publishers. Dordrecht, The Netherlands Cook

CRU (2015) University of East Anglia Climate Research Unit (CRU). CRU Datasets, [Internet]. Royal Meteorological Society, 1 September 2015. Available from https://cru.uea.ac.uk/

Mitchell TD, Jones PD (2005) An improved method of constructing a database of monthly climate observations and associated high-resolution grids. Int J Climatol 25:693–712.

2 Supplementary Figures and Tables

2.1 Supplementary Figures

Figure 1

a)

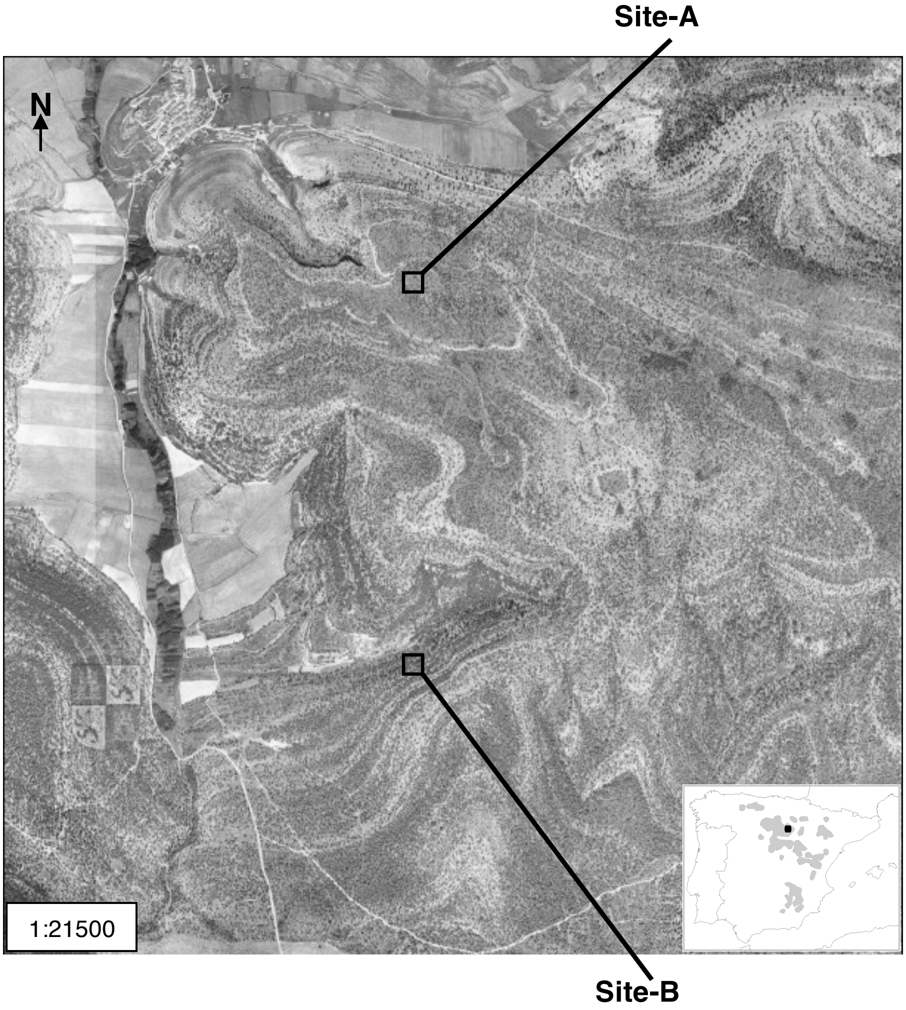

b)

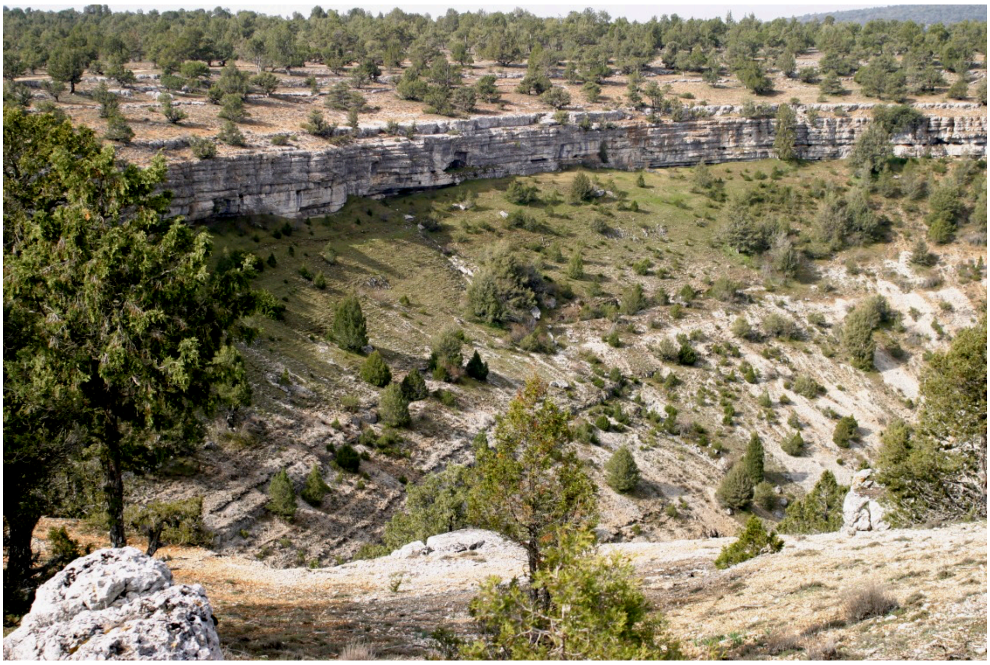

Site-A

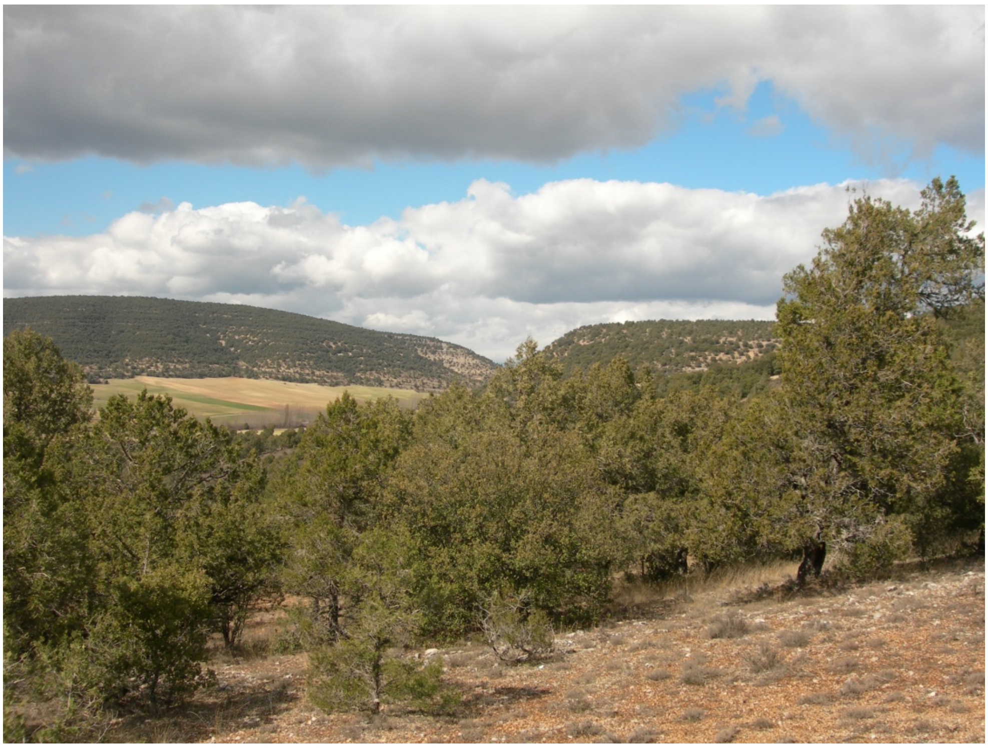

Site-B

Supplementary Figure 1. Study site characteristics. Study sites (a) location and (b) pictures. The map of the Iberian Peninsula in (a, bottom right) shows the distribution range of *Juniperus thurifera* in Spain (grey) and the location of the study sites (black) located at Cabrejas range, Soria, north-central Spain.

Figure 2

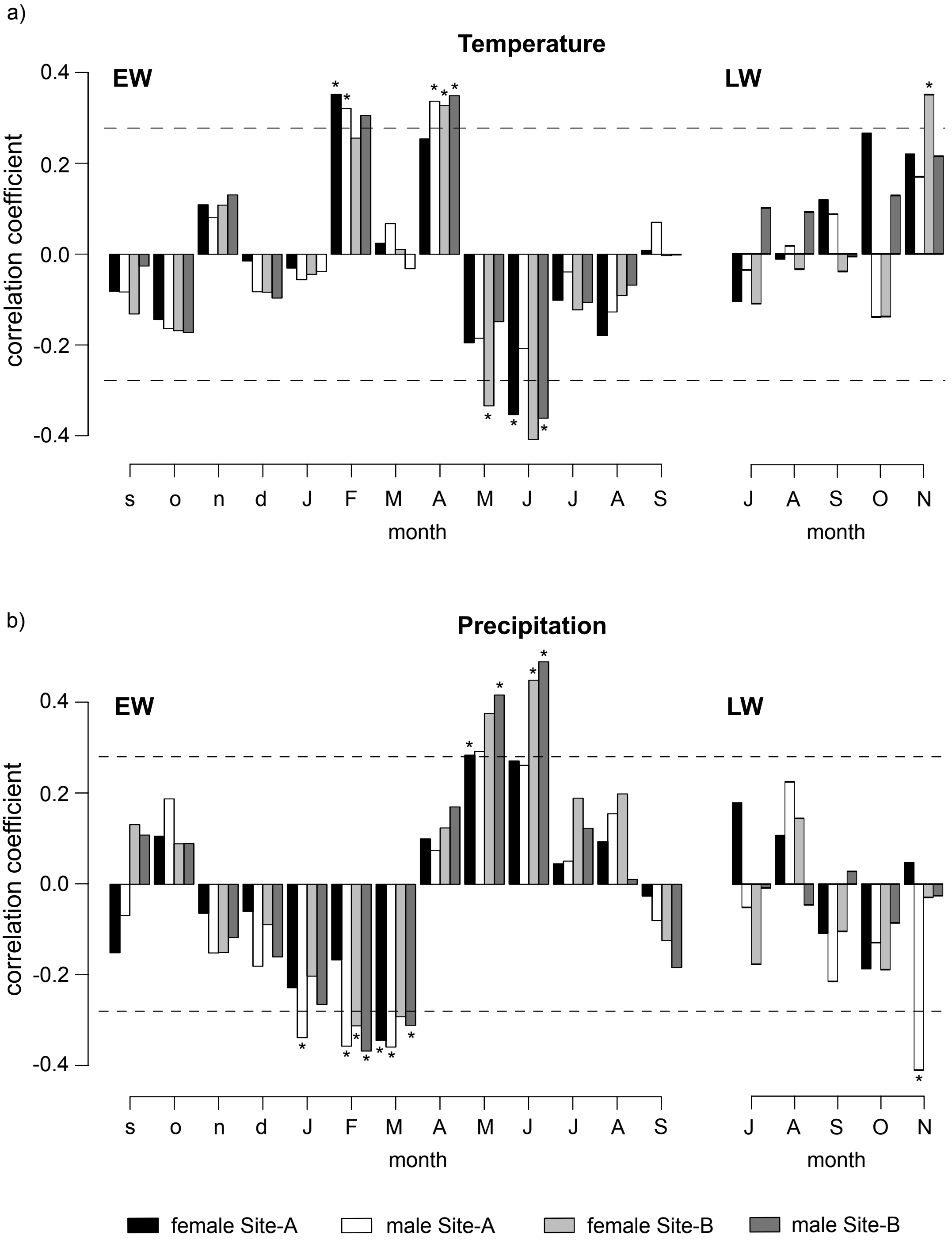

Supplementary Figure 2. Climate-growth relationship. Correlation coefficients calculated for the period 1961–2010 among the EW or LW growth indices for each site and sex combination and gridded monthly (a) mean temperature and (b) total precipitation. Months before and during the year of tree-ring formation are abbreviated by lower- and upper-case letters, respectively. Dashed lines indicate the 95% confidence boundary for significance levels of the Pearson correlation coefficients, and asterisks indicate significant (P<0.05) effect of climatic variables in accordance with the GLMM tests for each site and sex combination (see Supplementary Methods 1.2).

2.2 Supplementary Tables

Supplementary Table 1. Tree features. Mean and standard deviation (SD) for DBH, height and age of the sampled trees by each factor level. Mean values of tree features are compared with a *t* test, and no significant differences were found between and within the sexes and the sites.

		DBH (cm)					Height (m)				Age (yr)			
		**n**	**mean**	**SD**	***t***	***P***	**mean**	**SD**	***t***	***P***	**mean**	**SD**	***t***	***P***
Site-A	**male**	20	28.28	10.20	1.74	0.09	6.54	1.10	0.85	0.40	113.4	27.0	-0.70	0.47
	**female**	20	23.87	4.90			6.25	1.10			119.9	28.9		
Site-B	**male**	20	24.82	7.27	-0.90	0.40	6.18	1.20	-0.80	0.41	125.2	37.0	0.85	0.40
	**female**	20	26.79	7.33			6.50	1.30			116.5	27.4		
site	**Site-A**	40	26.07	8.23	0.16	0.88	6.39	1.10	0.21	0.83	116.6	27.8	-0.60	0.53
	**Site-B**	40	25.80	7.27			6.34	1.20			120.9	32.5		
sex	**male**	40	26.55	8.94	0.71	0.48	6.36	1.10	-0.10	0.96	119.3	32.5	0.16	0.87
	**female**	40	25.33	6.33			6.37	1.20			118.2	27.8		
